# Body weight misperception and academic performance in Chinese adolescents (2007–2022): the mediating role of loneliness

**DOI:** 10.3389/fpubh.2025.1665520

**Published:** 2026-01-13

**Authors:** Zeng-Bao Hu, Jin-Ying Huang, Stuart McDonald, Dan-Jie Jiang, Si-Jia Wang, Feng Wang, Qing-Hai Gong, Yi Lin

**Affiliations:** 1 Faculty of Humanities and Social Sciences, University of Nottingham Ningbo China, Ningbo, Zhejiang, China; 2 College of International Economics and Trade, Ningbo University of Finance and Economics, Ningbo, Zhejiang, China; 3 Ningbo Municipal Center for Disease Control and Prevention, Ningbo, Zhejiang, China

**Keywords:** body weight status, body weight misperception, academic performance, loneliness, mediation analysis, adolescents

## Abstract

**Introduction:**

Body weight misperception is increasingly prevalent among adolescents, yet the association between weight misperception and academic performances is unclear. This study aims to examine the association among weight misperception and academic performances among Chinese adolescents and estimate the mediating effect of loneliness on this association.

**Methods:**

This repeated cross-sectional study uses data of adolescents aged 13–19 years, collected from the Ningbo Youth Risk Behavior Surveys from 2007 to 2022. Data of anthropometric, demographic, weight perception, mood, and academic performance information were collected via self-reported questionnaires. Multivariate logit regression is used to investigate the association between weight misperception and academic performance. A generalized structural equation model is used to assess the mediating effect of loneliness on this association.

**Results:**

The sample sizes for each wave were 921, 909, 1,555, and 2,690. Adolescents with overestimation of body weight were significantly associated with higher odds of having poor academic performances for both girls (OR: 1.23, 95% CI: 1.03–1.46) and boys (OR: 1.45, 95% CI: 1.18–1.77). Specifically, body weight overestimation was significantly associated with higher odds of loneliness for girls (OR: 1.28, 95% CI: 1.05–1.56) and boys (OR: 1.40, 95% CI: 1.11–1.75), while loneliness was significantly associated with poorer academic performances for girls (OR: 1.34, 95% CI: 1.05–1.71) and boys (OR: 1.31, 95% CI: 1.09–1.59). Loneliness played a mediating role in the association between overestimation of body weight and poor academic performances, with indirect effects of 58.19 and 55.85% for girls (OR: 1.69, 95% CI: 1.14–2.24) and boys (OR: 1.81, 95% CI: 1.28–2.34), respectively.

**Discussion:**

Overestimation of body weight is associated with poorer academic performances in Chinese adolescents, mediated by loneliness. This finding suggests that family and school-based health education and psychological interventions, such as education on body image, targeted mental health consulting, mutual aid networks, fostering inclusive classroom, and family-school collaboration, should be encouraged to the well-being of adolescents.

## 1 Introduction

Adolescence is the critical transitional period that usually coincides with misperception of body weight (1, 2). Misperception of weight status among adolescents has become a globally pervasive public health problem (3–5). This is of particular concern as emerging studies begun to focus on the association between weight misperception and behavioral problems in adolescents (5, 6). However, the association between weight misperception and academic performances among adolescents, particularly the potential role of loneliness on this association, has not been well explored in the literature.

The adolescence period usually coincides with body image concerns. Dissatisfaction on body image is closely linked with misperception of body weight, and adolescents who self-perceived as overweight were prone to be dissatisfied with their body image (7). Dissatisfaction on body image was also found to be associated with greater loneliness among adolescents (8, 9). Particularly, the overestimation of weight status, as a typical body image concern among adolescents, could be closely associated with loneliness.

According to the Stigma Theory, stigma could make a person reduced “from a whole and usual person to a tainted, discounted one,” with social construction playing as the core in this process (10). Overweight and obese people often suffer negative social stereotypes such as being lazy and lacking self-discipline, as well as blame and discrimination from others (11). Therefore, individuals who overestimated their weight status may be more likely to avoid potential stigma by reducing social interaction, due to the widespread stigma attached to obesity (12). This psychological process is acknowledged as “weight stigma internalization” in literature, as individuals apply the weight-based societal stigma to themselves and therefore devalue themselves (13, 14).

Another important reason why overestimation of weight may be associated with loneliness is the mental health problems associated with it. According to the Body-Image Theory, adolescents’ perception of their bodies is shaped by social and cultural ideals characterized by thinness and muscularity (15). However, for adolescents who overestimated their weight, the gap between their perceived weight and their ideal weight may lead to body dissatisfaction and therefore the risk of various mental problems, including depressive and anxiety symptoms (16). These mental problems were closely linked to loneliness (17, 18).

Besides, loneliness during adolescence was shown to be associated with poor academic performances (19–21). According to the Positive and Negative Learning (PNL) Theory, students’ academic achievement is influenced by their study-related resources such as social support and self-efficacy (22). However, these resources could be undermined by the mental and emotional problems arising from loneliness, making loneliness linked to poor academic performances. In terms of self-efficacy, evidence suggested that loneliness was associated with reduced self-worth (19), and sleep and mental health problems (23), which may reduce the self-efficacy necessary for academic achievement. In terms of social support, loneliness was shown to be associated with reduced support networks from friends and family (24), which may lead to the absence of a supportive environment for adolescents to concentrate on study.

Given the important role of adolescents’ academic performances in predicting their achievement and health conditions in adulthood (25, 26), it is necessary to address the association between weight misperception and academic performances among adolescents. However, to the best of our knowledge, there is only two existing literature on this area, focusing on the US and Canada, respectively. These studies found that overweight perception was associated with poorer academic performances, independent of their actual weight status (27, 28). However, they did not further examine the role of specific factors, particularly mental health, play in this association. This has prevented the possibility of proposing targeted interventions to improve the well-being and academic achievement of adolescents. Additionally, loneliness is a common mental health issue with negative effect on mental and physical health, and is particularly likely to peak in adolescent years (29, 30). While exiting studies on adolescence multidimensional body image (e.g., body image dissatisfaction, weight misperception) mainly focused on its associations with behavioral problems (5, 6), while rarely examined it in relation to academic performances and loneliness.

Particularly, China serves as a good example to study the association between weight misperception and academic performances, because of its unique social and cultural context for adolescents. Like studies on Western adolescents, studies found that many Chinese adolescents have expressed dissatisfaction on body size (31, 32), and body weight misperception has become a common problem for adolescents in China (5, 6, 33). For instance, according to a recent survey in Guangzhou, China, among 5,734 students aged 8–12, 78.10% of the respondents showed different degree of body weight dissatisfaction (34).

Besides, the traditional Chinese culture has highly valued education, and obtaining diplomas of higher education has been the primary way to achieve career success and improve life quality for Chinese youth (35, 36). Therefore, educational stress has been common among Chinese school adolescents, and they face tougher academic competition than their Western peers (35–37). This has created a cultural and educational context in which Chinese adolescents experience simultaneous pressure from body weight concerns and academic pressure. The Chinese context could act as an amplifier, making the impact of body misperception on academic performances more pronounced and explicit. This has provided a valuable study opportunity to explore the link between adolescents’ weight misperception and academic performances.

Recent studies in China found that poor perceived appearance and overweight perception had negative impacts on adolescents’ academic achievement (38, 39). However, the specific relationship between weight misperception and academic performances in adolescents, and the effect of loneliness in this association, has not been studied in China, and this study aims to fill the gap in literature. This study could enrich the Positive and Negative Learning Theory and Weight Stigma Theory by suggesting the importance of body-related concerns and loneliness as study-related resources, and show the pathway through which mental and emotional issues arising from weight misperception translate into poor academic performances. Besides, this study has practical contribution by supporting targeted interventions to improve the well-being and academic achievement of adolescents.

Our research questions are “What is the association between weight misperception and academic performances among adolescents?” and “Does loneliness play a mediating effect on this association?.” Based on the above discussions on weight misperception, loneliness, academic performances, as well as the cultural and educational context for Chinese adolescents, we propose the following hypotheses:

*H1*: Weight misperception is negatively associated with academic performances among adolescents.

*H2*: Weight misperception is positively associated with loneliness among adolescents.

*H3*: Loneliness is negatively associated with academic performances among adolescents.

*H4*: Loneliness mediates the association between weight misperception and academic performances among adolescents.

## 2 Methodology and data

### 2.1 Study design and study population

This study uses repeated survey data from the Ningbo Youth Risk Behavior Survey (NYRBS). The NYRBS is an ongoing repeated cross-sectional survey, undertaken by the Ningbo Center for Disease Control and Prevention (CDC), designed to collect the health and risky behaviors of middle and high school adolescents in Ningbo, China. This present study included four repeated surveys in 2007, 2012, 2017, and 2022, and a multistage, stratified cluster sampling procedure was utilized to draw the target samples. At the first stage, 3 out of 10 districts in Ningbo were selected at random, representing one urban area, one urban–rural transitional area, and one rural area. At the second stage, target schools were randomly selected from the 3 districts based on school levels stratified by junior middle school, senior middle school, and vocational high school (2007, 2012 and 2017: 3:1:1 and 2022: 12:6:6). At the third stage, two classes were randomly selected from each selected school to conduct the survey, with the permission from school principals and administrations. There were 1,022, 1,204, 2,144, and 2,787 questionnaires collected in 2007, 2012, 2017, and 2022, respectively. According to previous studies, the estimated minimum sample size for a cross-sectional survey should be 196 (40, 41). The sample size for each survey wave in the NYRBS has substantially exceeded the level, ensuring the representativeness. Details of study design were reported previously in the literature (6).

**Figure 1:**
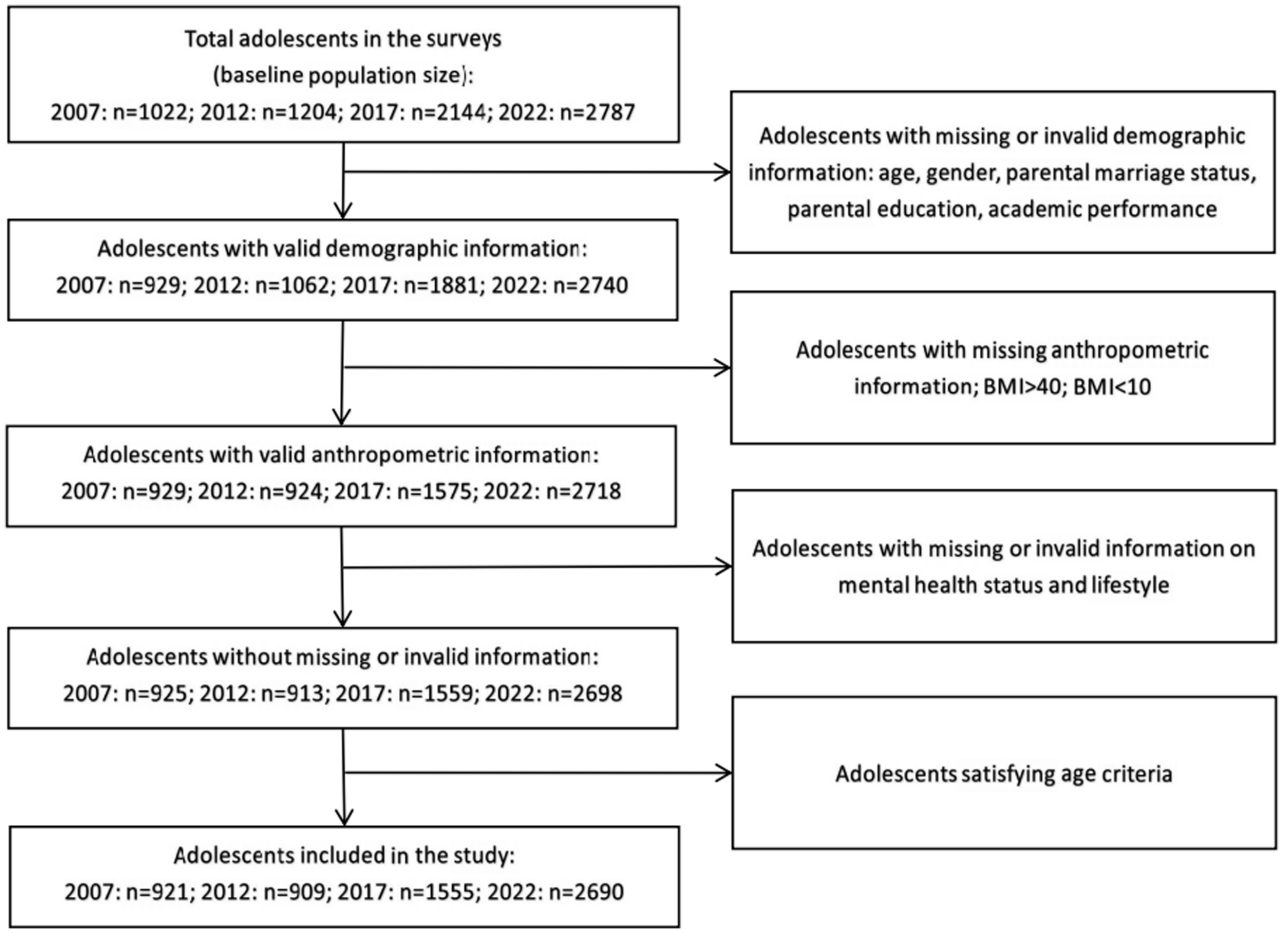
Flowchart of study population participating Ningbo youth risk behavior survey from 2007 to 2022.

The survey received ethics approval from the Ningbo CDC (No. 202011) and followed procedures consistent with the Declaration of Helsinki. Written informed consent was obtained from all participants, their parents or legal guardians and school officials. Verbal consent was witnessed and formally recorded. The inclusion criteria for the present study were: (1) adolescents born in Ningbo or had lived in Ningbo for at least one year; (2) aged between 13 and 19 years; (3) whose data on the demographic, anthropometric, mood, and academic performance information was available in the surveys; (4) had signed the consent to participate in the surveys; (5) had gained the informed consent of their parents or legal guardian for their participation in the surveys. The flowchart for data inclusion is presented as Figure 1. During the data cleaning, individuals who had missing or invalid values for any of the variables included in our analysis (i.e., weight misperception, academic performance, loneliness, BMI, age, parental marriage status, parental education status, frequencies of participating 60-min physical activities per week, consumption of junk food per week, and sleep duration overnight) were excluded. Therefore, the sample in the present study consists only of individuals with complete data on all relevant variables.

### 2.2 Questionnaires

The survey procedures were designed to ensure the anonymous and voluntary participation of adolescents. Adolescents were invited to complete anonymous self-reported paper questionnaires. Adolescents completed the questionnaires independently, without the presence of any teacher or parent but under the supervision of experienced public health specialists from the Ningbo CDC.

The design of the questionnaires was based on the US 1991–2015 Youth Risk Behavior Health Surveillance System and the WHO Global School-based Student Health Survey. The questionnaire covered the participants’ socio-economic and demographic characteristics, lifestyle, and physical and mental health status. The questionnaire was designed by Chinese public health specialists and was culturally and linguistically adapted for the Chinese adolescent context. Besides, the questionnaire was reviewed, revised, and approved after a pilot study by public health specialists (6, 41, 42). The design of the questionnaire was also used for research in other regions of Zhejiang Province in addition to Ningbo (42–44). Previous studies used this questionnaire have shown good reliability and validity in its measurement on the weight misperception, health behaviors, and mental health status of Chinese adolescents (6, 41–44). Public health specialists conducted quality control by thoroughly reviewing the submitted questionnaires. If there were any missing or misreported information, participants were encouraged, but were not obliged to re-complete the questionnaires.

### 2.3 Assessment of body weight status and weight perception

Weight status of adolescents was defined by their Body Mass Index (BMI) z-score classification determined by their BMI (kg/m2). Adolescents reported their information on weight (kg) and height (cm) based on their latest annual health check at school. BMI-for-age z-scores were then calculated to standardize the BMI values across sex. Based on these scores, adolescents were classified into 5 categories according to the classification provided by WHO (45): severe underweight (BMI-for-age<-3SD), underweight (−3SD ≤ BMI-for-age<-2SD), normal weight (−2SD ≤ BMI-for-age≤ + 1SD), overweight (+1SD < BMI-for-age≤ + 2SD), and obesity (BMI-for-age> + 2SD).

Self-perceived weight status was assessed by a self-reported question asking “How would you describe your current weight status?” Adolescents were required to choose from 5 options: “severe underweight,” “underweight,” “normal/ about the right weight,” “overweight,” and “obesity.”

Body weight misperception was assessed by comparing an adolescent’s perceived weight status and actual weight status (5, 6). Body weight misperception was categorized into 3 groups: overestimation (self-perceived weight status higher than actual weight classification), underestimation (self-perceived weight status lower than actual weight classification), and consistency (self-perceived status consistent to actual weight classification).

### 2.4 Assessment of academic performance

Academic performance was assessed based on adolescents’ self-reported response to the question “How would you describe your academic performance in your class?” with options of “poor,” “normal,” and “good.” To analyze the potential negative effect of weight misperception on academic performances, adolescents’ academic performance was classified into “poor” and “normal and good” in the present study.

### 2.5 Assessment of loneliness

In the survey, all adolescents were asked to answer a question of “Did you feel lonely in the past 12 months?” and given the option of the following 5 responses: “never,” “occasionally,” “sometimes,” “often,” and “always.” Since few adolescents answered “often” and “always,” and adolescents may feel ambiguous between “occasionally” and “sometimes,” loneliness was classified as “No” and “Yes” in the present study, where “Yes” indicates having experienced loneliness in the past 12 months, while “No” indicates not having experienced.

### 2.6 Statistical analysis

Descriptive statistics were reported as number and percentages (%) for categorical variables, and means and standard deviations (SD) for continuous variables. Differences in percentages and mean values across survey waves were analyzed using Chi-square test for categorical variables and Student’s t-test for continuous variables, respectively.

The logit model with robust standard errors was applied to assess the associations between weight misperception and loneliness, loneliness and academic performances, as well as weight misperception and academic performances in adolescents stratified by sex, controlling for demography and socio-economics status (SES) (age, parental marriage status, parental education status) and lifestyle (frequencies of participating 60-min physical activities per week, consumption of junk food per week, and sleep duration overnight). We used the logit model because the outcome variables (weight misperception, loneliness, academic performances) in our regressions were binary. The logit model is the widely used, and easy to interpret method for modeling a binary outcome (46). The data, containing four waves in 2007, 2012, 2017, and 2022, were treated as pooled cross-sectional samples.

Additionally, mediation analysis was performed using the generalized structural equation model with robust standard errors, controlling for the above-mentioned confounding factors (47). Poor academic performance was used as the dependent variable, weight misperception as the independent variable, and loneliness as the mediator variable. The bootstrapping technique was performed with 500 replications in the mediation analysis to construct bias-corrected 95% confidence intervals of the effect coefficients (47).

All results were considered as statistically significant at a two-tailed level of 0.05. Statistical analyses were performed using the STATA statistical software package version 17 (2021).

## 3 Results

### 3.1 Study population and characteristics

There were 921, 909, 1,555, and 2,690 adolescents included in 2007, 2012, 2017, and 2022, respectively (Table 1). There were significant differences in the percentages of demographic characteristics of adolescents across the four waves (*p* < 0.001 for all category groups). For continuous variables, the mean age and sleep duration overnight of adolescents decreased gradually across the four waves (*p* < 0.001). These indicate significant changes in the demographic characteristics and lifestyles among adolescents from 2007 to 2022.

**Table 1:** Characteristics of adolescents from 2007 to 2022.

Characteristics	Year				*p*
	2007 (*n* = 921)	2012 (*n* = 909)	2017 (*n* = 1,555)	2022 (*n* = 2,690)	
*N* (%)					
Sex					<0.001
Boys	511 (55.48)	383 (42.13)	728 (46.82)	1,361 (50.59)	
Girls	410 (44.52)	526 (57.87)	827 (53.18)	1,329 (49.41)	
Parental marriage					<0.001
Nuclear family	876 (95.11)	835 (91.86)	1,424 (91.58)	2,392 (88.92)	
Separated/ Single-parent family	45 (4.89)	74 (8.14)	131 (8.42)	298 (11.08)	
Paternal education					<0.001
Primary education or below	183 (19.87)	157 (17.27)	140 (9.00)	262 (9.74)	
Secondary education	644 (69.92)	599 (65.90)	1,087 (69.00)	1946 (72.34)	
Higher education (college or above degree)	94 (10.21)	153 (16.83)	328 (21.09)	482 (17.92)	
Maternal education					<0.001
Primary education or below	268 (29.10)	237 (26.07)	203 (13.05)	426 (15.84)	
Secondary education	597 (64.82)	550 (60.51)	1,065 (68.49)	1769 (65.76)	
Higher education (college or above degree)	56 (6.08)	122 (13.42)	287 (18.46)	495 (18.40)	
60-min physical activity (day/week)					<0.001
No	239 (25.95)	199 (21.89)	261 (16.78)	569 (21.15)	
1–2 days	295 (32.03)	312 (34.32)	352 (22.64)	738 (27.43)	
3–4 days	227 (24.65)	183 (20.13)	368 (23.67)	689 (25.61)	
5–7 days	160 (17.437)	215 (23.65)	574 (36.91)	694 (25.80)	
Junk food consumption (day/week)					<0.001
No	604 (65.58)	565 (62.16)	844 (54.28)	1,593 (59.22)	
1–2 days	276 (29.97)	297 (32.67)	606 (38.97)	975 (36.25)	
3–7 days	41 (4.45)	47 (5.17)	105 (6.75)	122 (4.54)	
Body weight status					<0.001
Severe Underweight	14 (1.52)	14 (1.54)	19 (1.22)	55 (2.04)	
Underweight	77 (8.36)	47 (5.17)	96 (6.17)	124 (4.61)	
Normal	763 (82.84)	782 (86.03)	1,291 (83.02)	2090 (77.70)	
Overweight	50 (5.43)	56 (6.16)	116 (7.46)	314 (11.67)	
Obesity	17 (1.85)	10 (1.10)	33 (2.12)	107 (3.98)	
Body weight perception					<0.001
Severe Underweight	58 (6.30)	48 (5.28)	81 (5.21)	110 (4.09)	
Underweight	212 (23.02)	197 (21.67)	288 (18.52)	444 (16.51)	
Normal	399 (43.32)	383 (42.13)	656 (42.19)	1,056 (39.26)	
Overweight	222 (24.10)	250 (27.50)	478 (30.74)	893 (33.20)	
Obesity	30 (3.26)	31 (3.41)	52 (3.34)	187 (6.95)	
Weight misperception					0.001
Consistency	459 (49.84)	428 (47.08)	765 (49.20)	1,325 (49.26)	
Over-estimation	233 (25.30)	258 (28.38)	450 (28.94)	841 (31.26)	
Under-estimation	229 (24.86)	223 (24.53)	340 (21.86)	524 (19.48)	
Poor academic performance					0.015
No	702 (76.22)	698 (76.79)	1,185 (76.21)	1957 (72.75)	
Yes	219 (23.78)	211 (23.21)	370 (23.79)	733 (27.25)	
Feel loneliness in past 12 months					0.003
No	218 (23.67)	166 (18.26)	341 (21.93)	647 (24.05)	
Yes	703 (76.33)	743 (81.74)	1,214 (78.07)	2043 (75.95)	
Mean (SD)					
Age (years)	16.16 (1.75)	16.14 (1.73)	15.83 (1.64)^a^^b^	15.89 (1.82)^a^^b^	<0.001
Sleep duration overnight (hours/day)	8.20 (1.16)	7.96 (1.41)	7.90 (1.53)^a^	7.61 (1.37)^a^^b^^c^	<0.001
Weight (kg)	52.32 (10.88)	52.49 (9.12)	54.48 (10.63)^a^^b^	56.74 (12.44)^a^^b^^c^	<0.001
Height (cm)	164.06 (8.84)	164.71 (8.31)	166.35 (8.54)^a^^b^	166.72 (8.54)^a^^b^	<0.001
BMI (kg/m2)	19.31 (2.98)	19.29 (2.69)	19.61 (3.11)	20.31 (3.64)^a^^b^^c^	<0.001

Comparisons between four waves were conducted using chi square test for category variables, and using oneway ANOVA for continuous variables.

a Mean values were significantly different from 2007 by oneway ANOVA with Bonferroni *Post-hoc*.

b Mean values were significantly different from 2012 by oneway ANOVA with Bonferroni *Post-hoc*.

c Mean values were significantly different from 2017 by oneway ANOVA with Bonferroni *Post-hoc*.

### 3.2 Body weight status and body weight misperception

The distribution of anthropometric characteristics, body weight status, and body weight misperception of adolescents exhibited significant trends from 2007 to 2022. Weight, height, and BMI of adolescents increased gradually from 2007 to 2022 (*p* < 0.001), particularly for the mean values in 2022, which were significantly higher than those in 2007 (Table 1). Regarding adolescents’ body weight status, the total proportion of severe underweight and underweight decreased from 9.88% in 2007 to 6.65% in 2022. In contrast, the total proportion of overweight and obesity increased from 7.28% in 2007 to 15.65% in 2022.

In terms of body weight misperception, around half of the adolescents kept correct self-perception of body weight, varying from 49.84 to 49.26% across waves. The proportion of adolescents overestimated their body weight increased from 25.30% in 2007 to 31.26% in 2022, while the proportion of those underestimated decreased from 24.86 to 19.48%.

**Figure 2:**
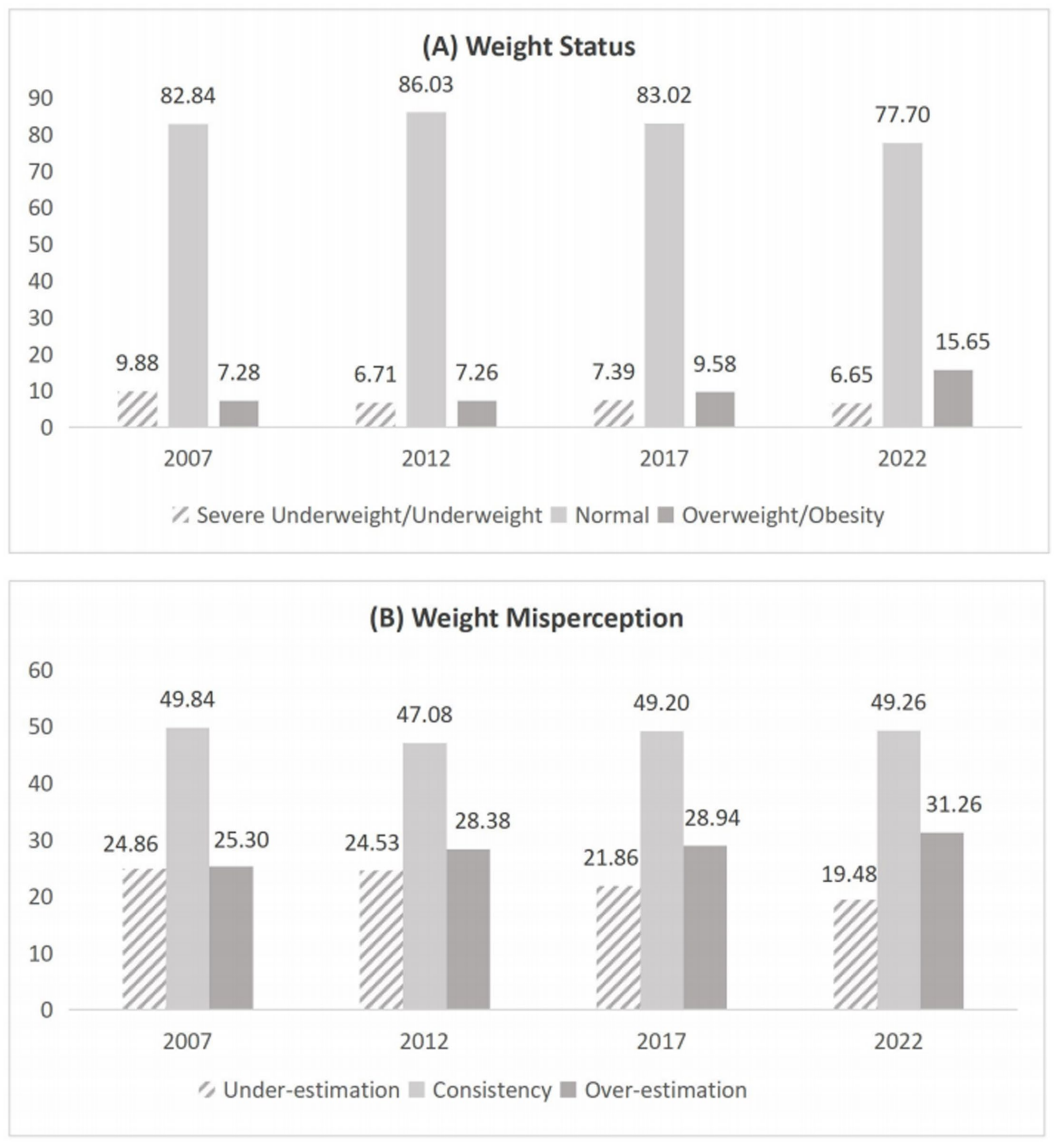
Trend of weight status and weight misperception among adolescents from 2007 to 2022. Charts in **(A)** shows the weight status among adolescents in 2007, 2012, 2017, and 2022. Charts in **(B)** shows the weight misperception status among adolescents in 2007, 2012, 2017, and 2022. Weight status was divided into three groups: severe underweight/underweight, normal weight, and overweight/obesity. Weight misperception was divided into three groups: under-estimation, consistency, and over-estimation. Numbers represent the proportions (%) of categories among total sample.

Figure 2 shows the trend of weight status and weight misperception among adolescents across the four survey waves. These results indicate that the prevalence of overweight and obesity, as well as overestimation of body weight, among adolescents, increased substantially from 2007 to 2022.

### 3.3 Academic performance and loneliness

From 2007 to 2022, there was a significant trend in the distribution of their academic performance status (*p* = 0.015), with the proportion of poor performance status increasing from 23.78% in 2007 to 27.25% in 2020 (Table 1). In each survey, 76.3, 81.74, 78.07, and 75.95% of the sample reported loneliness, respectively, with a significant trend across waves (*p* = 0.003). These results suggest consistently high prevalence of loneliness among adolescents between 2007 and 2022.

### 3.4 Associations among body weight perception, loneliness, and academic performances for adolescents

The multivariate logit regression analysis showed significant positive associations between adolescents’ overestimation of body weight and poor academic performances, between overestimation of body weight and loneliness, and between loneliness and poor academic performances, for both sexes, after controlling for confounding demographic and lifestyle factors (Table 2). No significant association was observed between underestimation of body weight and poor academic performances for both sexes. Details regarding the control variables’ coefficients can be found in the Supplementary material.

**Table 2:** Associations between weight misperception, poor academic performance, and loneliness in adolescents from 2007 to 2022.

Outcome variable	Predictor variable	Category	Weight over-estimation^a^			
			Girls *n* = 3,092		Boys *n* = 2,983	
			OR	95% CI	OR	95% CI
Poor academic performance	Over-estimation of weight	No	1		1	
		Yes	1.23	1.03, 1.46	1.45	1.18, 1.77
Loneliness	Over-estimation of weight	No	1		1	
		Yes	1.28	1.05, 1.56	1.40	1.11, 1.75
Poor academic performance	Loneliness	No	1		1	
		Yes	1.34	1.05, 1.71	1.31	1.09, 1.59

Outcome variable	Predictor variable	Category	Weight under-estimation^b^			
			Girls *n* = 3,092		Boys *n* = 2,983	
			OR	95% CI	OR	95% CI
Poor academic performance	Under-estimation of weight	No	1		1	
		Yes	0.84	0.65, 1.10	0.92	0.77, 1.10
Loneliness	Under-estimation of weight	No	1		1	
		Yes	1.48	1.10, 1.99	1.02	0.85, 1.22
Poor academic performance	Loneliness	No	1		1	
		Yes	1.34	1.05, 1.71	1.31	1.09, 1.59

OR, odds ratio; CI, confidence interval.

Regressions are conducted using the Logistic model, with controlling for age, parental marriage status, parental education status, frequencies of participating 60-min physical activities per week, consumption of junk food per week, sleep duration overnight, and survey wave.

a This section focuses on the associations between overestimation of weight, poor academic performance, and loneliness.

b This section focuses on the associations between underestimation of weight, poor academic performance, and loneliness.

**Figure 3:**
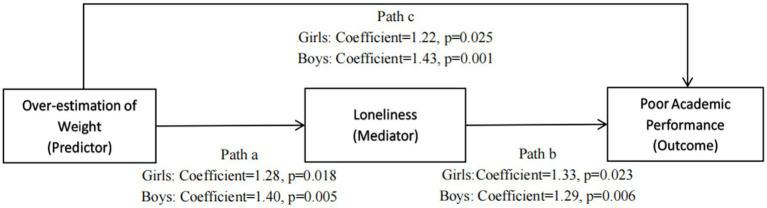
Graphical representation of the mediation analysis model. Regression coefficients derived from the structural equation model are displayed as odds ratio. Confounding factors of age, parental marriage status, parental education status, frequencies of participating 60-min physical activities per week, consumption of junk food per week, and survey wave were controlled for Bootstrapping with 500 replications was performed in the mediation analysis.

Figure 3 graphically depicts the mediating effect of loneliness in the association between overestimation of body weight and poor academic performances, and the regression coefficients from the generalized structural equation model. A significant association between adolescents’ overestimation of body weight and poor academic performances (path c) was found in both boys and girls. The associations between overestimation of body weight and loneliness (path a), and between loneliness and poor academic performances (path b) were also significant for both sexes. These indicate that adolescents, both boys and girls, who overestimated their body weight status were more likely to feel lonely and have poor academic performances.

The total, direct, and indirect effects were statistically significant in both sexes, indicating significant mediating effect of loneliness on overestimation of body weight and poor academic performances (Table 3). Specifically, the direct effects of overestimation of body weight on poor academic performances were 41.81% for adolescent girls and 44.15% for adolescent boys, respectively, in the present study. The indirect effects of overestimation of body weight via loneliness on poor academic performances were 58.19% for girls and 55.85% for boys. Overall, the results suggest a significant association between overestimation of body weight and poor academic performances among adolescents, and the association was mediated by loneliness.

**Table 3:** Results of the structural equation model: the total, direct, and indirect (via loneliness) effects of overestimation of weight on poor academic performances.

Effect	Mediation path	Girls			Boys		
		OR	95% CI	Effect size	OR	95% CI	Effect size
Direct effect^a^	c in Figure 2	1.22	1.01, 1.42^*^	41.81%	1.43	1.14, 1.72^*^	44.15%
Indirect effect^b^	ab in Figure 2	1.69	1.14, 2.24^*^	58.19%	1.81	1.28, 2.34^*^	55.85%
Total effect^c^	c + ab in Figure 2	2.91	2.31, 3.50^*^		3.23	2.62, 3.84^*^	

OR, odds ratio; CI, confidence interval.

Bootstrapping with 500 replications was performed in the mediation analysis to construct bias-corrected 95% CIs.

a Direct effect: overestimation of body weight on poor academic performances.

b Indirect effect (via loneliness): overestimation of body weight on poor academic performances.

c Total effect: overestimation of body weight on poor academic performances.

* *p* < 0.05.

## 4 Discussion

### 4.1 Discussion of findings

This study investigated the association between body weight misperception and academic performances in adolescents, as well as the mediating effect of loneliness in this association. This study found that overestimation of body weight was significantly associated with poor academic performances among adolescents. This association was significantly mediated by loneliness. The indirect effect of overestimation of body weight through loneliness on poor academic performances presented considerable proportions of 58.19% for girls and 55.85% for boys. However, underestimation of body weight was not significantly associated with poor academic performances.

Our findings could be explained by integrating the Life Skills Transfer (LST) Model with the Positive Youth Development (PYD) framework. The LST Model suggested that the beliefs and skills people obtained in one domain could be transferred into other domains and therefore influence their performances in these areas (48). However, for adolescents who overestimated their body weight, a negative transfer may occur: their emotional and mental problems, such as loneliness, in relation to weight misperception may be transferred into the academic area, influencing their academic performances. This process of negative transfer also aligns with the PYD framework. According to the PYD framework, adolescents have developmental plasticity, and characteristics such as competence, confidence, and connection are important assets in the health and positive development of adolescents (49). However, the overestimation of body weight could damage confidence through dissatisfaction on body image and therefore reduce connections with families and friends. The weakened confidence and connection may foster loneliness and ultimately undermine adolescents’ competence in academics. Therefore, a negative association between overestimation of weight and academic performances could be established.

The above-mentioned theoretical grounding linking weight misperception, loneliness, and poor academic performances, are further supported by our empirical results. First, this study showed a positive association between overestimation of body weight and poor academic performances among Chinese adolescents. According to studies in the United States and Canada, overweight perception was associated with poorer academic performances (27, 28). The present study further showed that in addition to overweight perception, overestimation of body weight was also associated with poorer academic performances in adolescents.

Second, the mediation analyses in the present study further suggested that loneliness played as a mediator on the association between overestimation of body weight and poorer academic performances. During adolescence, peer acceptance begins to play an increasingly important role in adolescents’ life (50), and body weight was reported as an important influencing factor of peer acceptance (51). Adolescents’ dissatisfaction on body shape and weight may invoke feelings of loneliness, which has been supported by studies in Russia (8), the United States (9), and China (52). This is closely connected with the internalization of weight stigma (53–56).

Third, the study showed that loneliness was associated with poorer academic performances in adolescents. According to a recent study in the United Kingdom, loneliness could negatively influence adolescents’ academic performances by lowering their self-worth (19). Another study revealed that support from friendship was important in buffering the negative impact of loneliness on adolescents’ academic performances (24). Loneliness was also found to be associated with poor sleep quality (23), depression and anxiety (57, 58), and internet gaming disorder (59). Given that mental health was found to be important for academic achievement among adolescents (60), mental problems associated with loneliness might be another channel linking loneliness and poor academic performances.

The indirect effect of loneliness was shown to be higher in girls (58.19%) than in boys (55.85%). In other words, loneliness played a greater role in mediating the association between overestimation of body weight and poor academic performances in adolescent girls than boys. This difference could be attributed to several possible reasons. First, the dominant socially preferred body shapes for boys and girls are different, which is thinness for females and muscularity for males. Hence, adolescent girls may have higher levels of body weight dissatisfaction than boys under such norms (61, 62). Second, although both boys and girls may be influenced by the “thin-ideal” images on media, the media may have greater thinness pressure on girls. This is because the media could exert an indirect impact on girls’ ideal body image by influencing boys’ expectations and evaluations of girls’ body images (63). Third previous literature revealed that adolescent girls had a higher risk of experiencing weight-related relational bullying than boys (64). Therefore, loneliness may had a stronger mediating effect on the association between overestimation of body weight and academic performance for girls than boys.

It is important to note that Chinese adolescents were raised in a cultural and educational environment distinct from that in Western countries, which may have had important implications on the association between weight misperception and academic performances. For example, a recent study found that Chinese adolescents preferred friendships with peers who performed better in academics and avoided friendships with academically disadvantaged peers (65). Additionally, the absence of siblings was believed to affect the personality development of adolescents (66). Hence, China’s one-child policy may also had interacted with adolescents’ academic performances and loneliness, which may be left for future research.

In addition, this study found that misperception of body weight has become a pervasive problem among Chinese adolescents. Over half of the adolescents misperceived their body weight status in each survey wave, with overestimation of weight being increasingly prevalent. This might be explained by the interaction of social and cultural context during the period. The rise of social media in China may have contributed to the body weight dissatisfaction for adolescents by fostering a culture of appearance comparison that could encourage overestimation of body weight (67). Additionally, the increasing influence of Western culture and media may also contribute to the overweight misperception of Chinese adolescents, as previous literature suggested that Western culture influence was significant associated with adolescents’ weight dissatisfaction (68).

Speaking more broadly, our findings contribute to an emerging literature studying the association between weight misperception and academic performances in adolescents. We identify a mediating effect in the form of loneliness, which played as a mediator on the association between overestimation of body weight and academic performance and has not been previously identified in the literature. Another strength of this study is that we used data from a 15-year study with 4 repeated surveys covering demographic, socio-economic, lifestyle, and mental health information of participants. However, some limitations should be noticed. First, the data was collected in self-administered anonymous surveys, which might be subject to respondent memories and bias. Specifically, the data for some sensitive variables, such as weight, height, lifestyle, and mental health, relied on the self-reporting of participants, which might be affected by social desirability bias, recall bias, and misreporting. Second, we only considered the mediating effect of loneliness due to the design of questionnaires. The association between weight misperception and academic performances may also be mediated by other negative mood characteristics or mental problems (e.g., depression, sadness, stress, anxiety). Third, our study was based on a cross-sectional design and therefore could not establish a causality between weight misperception and academic performances among adolescents. Fourth, our study was only conducted in Ningbo, China, hence our results may not be generalized to other parts of China. Fifth, due to the design of the questionnaires, our study did not include certain individual and socio-economic characteristics of adolescents (e.g., intelligence, learning behaviors, socio-economic status of family) that may affect the associations between adolescents’ body weight misperception, loneliness, and academic performances. Therefore, some omitted variables may affect the accuracy of the associations.

Future well-designed longitudinal studies, including comprehensive (e.g., multi-item scales) and objective measurement, and the inclusion of more detailed socio-economic and individual characteristics of adolescents, are needed to confirm the causality between weight misperception and poor academic performances. Additionally, future studies may consider exploring the roles of other psychological status, such as depression, sadness, anxiety, stress, on the association between weight misperception and academic performances. Furthermore, multi-center studies that involve a larger, more diverse and more representative population should be considered in the future, given the significant regional heterogeneity in socio-economic and cultural geographical characteristics in China. Besides, with recent literature noticing the importance of loneliness prevention for adolescents within school environment (69), future studies may explore the effectiveness of the interventions in reducing the negative effects of weight misperception on academic performances.

### 4.2 Implications

Our findings reveal that adolescents who overestimated their body weight status were associated with poorer academic performances, while such an association was mediated by the loneliness associated with overestimation of body weight. Our findings could provide policymakers with important insights into the well-being of adolescents with weight misperception. First, family- and school-based health education programs on awareness of obesity and weight perception are needed. Particularly, adolescents should be educated to correctly understand body image and to avoid weight-related labeling and discrimination, which could help foster an inclusive classroom that reduces weight-related stigma. There have been weight-related anti-discrimination programs and interventions carried out in some Western countries (70). Similar interventions should also be considered by schools and government in China. These interventions should be tailored to fit the Chinese culture and context, based on the existing effective programs. Second, the schools may consider recruiting specialists in adolescent psychology and education to provide psychological consulting services and tailored support strategies for adolescents who overestimated their body weight status, to address their feelings of loneliness as well as other mental problems associated with their weight misperception. Third, mutual support groups among adolescents, led by peer support specialists, should be encouraged at schools. These groups would provide a safe space for adolescents with body image concerns to provide mutual emotional support and reduce isolation. Fourth, cooperation between family education and school education should be promoted to foster family support and thereby improve the well-being of adolescents who suffer from weight misperception. This could include joint workshops for parents and teachers to create a consistent and supportive environment across home and school, particularly for adolescents suffering weight misperception. Finally, the impacts of the above-mentioned interventions should be evaluated regularly through rigorous and scientific measures to ensure the effectiveness of the interventions.

## 5 Conclusion

This present study found an increasing trend in the prevalence of weight overestimation and a decreasing trend in weight underestimation among Chinese adolescents from 2007 to 2022. Overestimation of body weight was significantly associated with poorer academic performances in adolescents, with loneliness identified as a mediating factor in this association. Our findings could assist school management and public health specialists for improvement of strategies for health education in reducing the negative psychological effects and improvement of adolescents’ mental health well-being and academic performance.

## Data Availability

The data is not publicly available due to privacy or ethical restrictions. If there is a reasonable request, it can be obtained from the corresponding authors.
