# Supplementary material for: Body weight misperception and academic performance in Chinese adolescents (2007–2022): the mediating role of loneliness
Source: Front Public Health. 2026 Jan 13;13:1665520. doi: 10.3389/fpubh.2025.1665520 (PMC12835246; doi:10.3389/fpubh.2025.1665520)
Supplement: Supplementary file 1 [file Table_1.DOCX]

Supplementary Material

**Supplementary Table 1.** Associations between over-estimation of weight and poor academic performance for girls and boys.

| **Outcome Variable:**  **Poor Academic Performance** | **Description** | Girls n=3092^a^ | | | Boys n=2983^b^ | | |
| --- | --- | --- | --- | --- | --- | --- | --- |
|  |  | OR | 95% CI | P | OR | 95% CI | P |
| Over-estimation of Weight | No | 1 |  |  | 1 |  |  |
|  | Yes | 1.23 | 1.03, 1.46 | 0.021 | 1.45 | 1.18, 1.77 | <0.001 |
| Age | Years | 0.99 | 0.94, 1.04 | 0.813 | 0.99 | 0.95, 1.04 | 0.778 |
| Parental Marriage Status | Separated/ Single-parent family | 1 |  |  | 1 |  |  |
|  | Nuclear family | 0.99 | 0.74, 1.32 | 0.925 | 0.81 | 0.61, 1.08 | 0.159 |
| Paternal Education | Primary education or below | 1 |  |  | 1 |  |  |
|  | Secondary education | 1.07 | 0.81, 1.41 | 0.644 | 0.86 | 0.67, 1.10 | 0.229 |
|  | Higher education (college or above degree) | 1.02 | 0.68, 1.51 | 0.939 | 0.75 | 0.52, 1.08 | 0.123 |
| Maternal Education | Primary education or below | 1 |  |  | 1 |  |  |
|  | Secondary education | 0.90 | 0.71, 1.14 | 0.365 | 0.78 | 0.63, 0.97 | 0.022 |
|  | Higher education (college or above degree) | 0.61 | 0.42, 0.89 | 0.011 | 0.51 | 0.36, 0.74 | <0.001 |
| 60-min Physical Activity | No | 1 |  |  | 1 |  |  |
|  | 1-2 days/week | 0.81 | 0.65, 1.01 | 0.059 | 0.82 | 0.64, 1.07 | 0.139 |
|  | 3-4 days/week | 0.59 | 0.45, 0.75 | <0.001 | 0.76 | 0.59, 0.99 | 0.041 |
|  | 5-7 days/week | 0.69 | 0.53, 0.90 | 0.007 | 0.87 | 0.68, 1.12 | 0.278 |
| Junk food Consumption | No | 1 |  |  | 1 |  |  |
|  | 1-2 days/week | 1.06 | 0.88, 1.27 | 0.556 | 1.35 | 1.13, 1.61 | 0.001 |
|  | 3-7 days/week | 2.31 | 1.60, 3.35 | <0.001 | 1.73 | 1.24, 2.42 | 0.001 |
| Sleep duration overnight | Hours/day | 0.91 | 0.85, 0.98 | 0.008 | 0.96 | 0.90, 1.02 | 0.201 |
| Survey Year | 2007 | 1 |  |  | 1 |  |  |
|  | 2012 | 1.26 | 0.90, 1.76 | 0.174 | 0.90 | 0.66, 1.22 | 0.498 |
|  | 2017 | 1.16 | 0.84, 1.59 | 0.363 | 1.10 | 0.85, 1.42 | 0.479 |
|  | 2022 | 1.69 | 1.26, 2.25 | <0.001 | 1.00 | 0.79, 1.26 | 0.976 |
| **Note:**  a The Pseudo R^2^ is 0.03, the AIC is 3227.13, and the BIC is 3329.75.  b The Pseudo R^2^ is 0.02, the AIC is 3518.82, and the BIC is 3620.53. | | | | | | | |

**Supplementary Table 2.** Associations between under-estimation of weight and poor academic performance for girls and boys.

| **Outcome Variable:**  **Poor Academic Performance** | **Description** | Girls n=3092^a^ | | | Boys n=2983^b^ | | |
| --- | --- | --- | --- | --- | --- | --- | --- |
|  |  | OR | 95% CI | P | OR | 95% CI | P |
| Under-estimation of Weight | No | 1 |  |  | 1 |  |  |
|  | Yes | 0.84 | 0.65, 1.10 | 0.204 | 0.92 | 0.77, 1.10 | 0.342 |
| Age | Years | 1.00 | 0.94, 1.05 | 0.853 | 0.99 | 0.95, 1.04 | 0.820 |
| Parental Marriage Status | Separated/ Single-parent family | 1 |  |  | 1 |  |  |
|  | Nuclear family | 0.99 | 0.73, 1.32 | 0.938 | 0.81 | 0.61, 1.08 | 0.158 |
| Paternal Education | Primary education or below | 1 |  |  | 1 |  |  |
|  | Secondary education | 1.05 | 0.80, 1.38 | 0.728 | 0.85 | 0.66, 1.10 | 0.212 |
|  | Higher education (college or above degree) | 1.00 | 0.68, 1.49 | 0.982 | 0.74 | 0.51, 1.08 | 0.118 |
| Maternal Education | Primary education or below | 1 |  |  | 1 |  |  |
|  | Secondary education | 0.90 | 0.71, 1.14 | 0.398 | 0 .77 | 0.63, 0.96 | 0.019 |
|  | Higher education (college or above degree) | 0.61 | 0.42, 0.90 | 0.012 | 0.52 | 0.36, 0.75 | <0.001 |
| 60-min Physical Activity | No | 1 |  |  | 1 |  |  |
|  | 1-2 days/week | 0.80 | 0.64, 1.00 | 0.049 | 0.82 | 0.63, 1.06 | 0.124 |
|  | 3-4 days/week | 0.59 | 0.46, 0.75 | <0.001 | 0.76 | 0.59, 0.99 | 0.040 |
|  | 5-7 days/week | 0.69 | 0.53, 0.90 | <0.001 | 0.86 | 0.67, 1.09 | 0.216 |
| Junk food Consumption | No | 1 |  |  | 1 |  |  |
|  | 1-2 days/week | 1.05 | 0.87, 1.26 | 0.623 | 1.35 | 1.13, 1.60 | 0.001 |
|  | 3-7 days/week | 2.31 | 1.60, 3.34 | <0.001 | 1.72 | 1.23, 2.39 | 0.001 |
| Sleep duration overnight | Hours/day | 0.91 | 0.84, 0.97 | 0.006 | 0.96 | 0.90, 1.02 | 0.190 |
| Survey Year | 2007 | 1 |  |  | 1 |  |  |
|  | 2012 | 1.27 | 0.91, 1.77 | 0.165 | 0.91 | 0.67, 1.23 | 0.537 |
|  | 2017 | 1.17 | 0.85, 1.61 | 0.328 | 1.10 | 0.85, 1.42 | 0.480 |
|  | 2022 | 1.71 | 1.28, 2.28 | <0.001 | 1.00 | 0.79, 1.26 | 0.990 |
| **Note:**  a The Pseudo R^2^ is 0.03, the AIC is 3230.71, and the BIC is 3333.33.  b The Pseudo R^2^ is 0.02, the AIC is 3530.17, and the BIC is 3632.18. | | | | | | | |

**Supplementary Table 3.** Associations between over-estimation of weight and loneliness for girls and boys.

| **Outcome Variable:**  **Loneliness** | **Description** | Girls n=3092^a^ | | | Boys n=2983^b^ | | |
| --- | --- | --- | --- | --- | --- | --- | --- |
|  |  | OR | 95% CI | P | OR | 95% CI | P |
| Over-estimation of Weight | No | 1 |  |  | 1 |  |  |
|  | Yes | 1.28 | 1.05, 1.56 | 0.016 | 1.40 | 1.11, 1.75 | 0.004 |
| Age | Years | 1.15 | 0.99, 1.11 | 0.114 | 1.11 | 1.06, 1.17 | <0.001 |
| Parental Marriage Status | Separated/ Single-parent family | 1 |  |  | 1 |  |  |
|  | Nuclear family | 0.58 | 0.40, 0.85 | 0.004 | 0.45 | 0.31, 0.64 | <0.001 |
| Paternal Education | Primary education or below | 1 |  |  |  |  |  |
|  | Secondary education | 0.85 | 0.62, 1.17 | 0.321 | 0.94 | 0.71, 1.23 | 0.651 |
|  | Higher education (college or above degree) | 0.98 | 0.64, 1.50 | 0.916 | 0.87 | 0.60, 1.25 | 0.442 |
| Maternal Education | Primary education or below | 1 |  |  | 1 |  |  |
|  | Secondary education | 1.04 | 0.80, 1.36 | 0.778 | 0.80 | 0.64, 1.01 | 0.058 |
|  | Higher education (college or above degree) | 0.80 | 0.54, 1.18 | 0.256 | 0.72 | 0.52, 1.02 | 0.062 |
| 60-min Physical Activity | No | 1 |  |  | 1 |  |  |
|  | 1-2 days/week | 1.00 | 0.77, 1.29 | 0.975 | 1.10 | 0.83, 1.44 | 0.509 |
|  | 3-4 days/week | 0.86 | 0.65, 1.13 | 0.281 | 1.12 | 0.86, 1.48 | 0.401 |
|  | 5-7 days/week | 0.77 | 0.58, 1.03 | 0.076 | 0.77 | 0.60, 1.00 | 0.046 |
| Junk food Consumption | No | 1 |  |  | 1 |  |  |
|  | 1-2 days/week | 1.06 | 0.86, 1.29 | 0.601 | 1.12 | 0.94, 1.35 | 0.202 |
|  | 3-7 days/week | 1.11 | 0.70, 1.77 | 0.656 | 1.34 | 0.93, 1.93 | 0.120 |
| Sleep duration overnight | Hours/day | 0.81 | 0.75, 0.87 | <0.001 | 0.85 | 0.80, 0.90 | <0.001 |
| Survey Year | 2007 | 1 |  |  |  |  |  |
|  | 2012 | 1.05 | 0.74, 1.50 | 0.779 | 1.55 | 1.13, 2.13 | 0.007 |
|  | 2017 | 0.94 | 0.68, 1.31 | 0.716 | 1.28 | 0.98, 1.66 | 0.068 |
|  | 2022 | 0.79 | 0.58, 1.07 | 0.126 | 0.98 | 0.78, 1.24 | 0.859 |
| **Note:**  a The Pseudo R^2^ is 0.02, the AIC is 2815.93, and the BIC is 2918.55.  b The Pseudo R^2^ is 0.04, the AIC is 3439.15, and the BIC is 3541.16. | | | | | | | |

**Supplementary Table 4.** Associations between under-estimation of weight and loneliness for girls and boys.

| **Outcome Variable:**  **Loneliness** | **Description** | Girls n=3092^a^ | | | Boys n=2983^b^ | | |
| --- | --- | --- | --- | --- | --- | --- | --- |
|  |  | OR | 95% CI | P | OR | 95% CI | P |
| Under-estimation of Weight | No | 1 |  |  | 1 |  |  |
|  | Yes | 1.48 | 1.10, 1.99 | 0.009 | 1.02 | 0.85, 1.22 | 0.852 |
| Age | Years | 1.06 | 1.00, 1.12 | 0.060 | 1.12 | 1.06, 1.17 | <0.001 |
| Parental Marriage Status | Separated/ Single-parent family | 1 |  |  | 1 |  |  |
|  | Nuclear family | 0.58 | 0.10, 0.83 | 0.003 | 0.45 | 0.32, 0.64 | <0.001 |
| Paternal Education | Primary education or below | 1 |  |  |  |  |  |
|  | Secondary education | 0.84 | 0.61, 1.16 | 0.297 | 0.93 | 0.71, 1.22 | 0.611 |
|  | Higher education (college or above degree) | 0.98 | 0.64, 1.50 | 0.922 | 0.86 | 0.60, 1.24 | 0.431 |
| Maternal Education | Primary education or below | 1 |  |  | 1 |  |  |
|  | Secondary education | 1.04 | 0.79, 1.36 | 0.781 | 0.80 | 0.63, 1.00 | 0.051 |
|  | Higher education (college or above degree) | 0.78 | 0.52, 1.15 | 0.211 | 0.73 | 0.52, 1.02 | 0.065 |
| 60-min Physical Activity | No | 1 |  |  | 1 |  |  |
|  | 1-2 days/week | 0.98 | 0.76, 1.28 | 0.896 | 1.09 | 0.83, 1.43 | 0.535 |
|  | 3-4 days/week | 0.86 | 0.65, 1.14 | 0.297 | 1.12 | 0.86, 1.48 | 0.400 |
|  | 5-7 days/week | 0.78 | 0.59, 1.04 | 0.083 | 0.76 | 0.59, 0.98 | 0.036 |
| Junk food Consumption | No | 1 |  |  | 1 |  |  |
|  | 1-2 days/week | 1.05 | 0.86, 1.28 | 0.646 | 1.12 | 0.94, 1.34 | 0.211 |
|  | 3-7 days/week | 1.07 | 0.67, 1.73 | 0.766 | 1.32 | 0.92, 1.91 | 0.135 |
| Sleep duration overnight | Hours/day | 0.80 | 0.75, 0.86 | <0.001 | 0.84 | 0.80, 0.90 | <0.001 |
| Survey Year | 2007 | 1 |  |  | 1 |  |  |
|  | 2012 | 1.03 | 0.72, 1.47 | 0.857 | 1.56 | 1.13, 2.14 | 0.006 |
|  | 2017 | 0.95 | 0.68, 1.31 | 0.739 | 1.28 | 0.98, 1.66 | 0.066 |
|  | 2022 | 0.81 | 0.59, 1.10 | 0.181 | 0.99 | 0.78, 1.25 | 0.912 |
| **Note:**  a The Pseudo R^2^ is 0.03, the AIC is 2814.55, and the BIC is 2917.17.  b The Pseudo R^2^ is 0.04, the AIC is 3447.87, and the BIC is 3549.88. | | | | | | | |

**Supplementary Table 5.** Associations between loneliness and poor academic performance for girls and boys.

| **Outcome Variable:**  **Poor Academic Performance** | **Description** | Girls n=3092^a^ | | | Boys n=2983^b^ | | |
| --- | --- | --- | --- | --- | --- | --- | --- |
|  |  | OR | 95% CI | P | OR | 95% CI | P |
| Loneliness | No | 1 |  |  | 1 |  |  |
|  | Yes | 1.34 | 1.05, 1.71 | 0.017 | 1.31 | 1.09, 1.59 | 0.005 |
| Age | Years | 1.00 | 0.94, 1.05 | 0.867 | 0.99 | 0.94, 1.04 | 0.655 |
| Parental Marriage Status | Separated/ Single-parent family | 1 |  |  | 1 |  |  |
|  | Nuclear family | 1.00 | 0.75, 1.35 | 0.978 | 0.84 | 0.63, 1.13 | 0.253 |
| Paternal Education | Primary education or below | 1 |  |  |  |  |  |
|  | Secondary education | 1.06 | 0.80, 1.39 | 0.690 | 0.85 | 0.66, 1.10 | 0.218 |
|  | Higher education (college or above degree) | 1.00 | 0.68, 1.49 | 0.987 | 0.75 | 0.52, 1.08 | 0.124 |
| Maternal Education | Primary education or below | 1 |  |  | 1 |  |  |
|  | Secondary education | 0.90 | 0.71, 1.14 | 0.381 | 0.78 | 0.63, 0.97 | 0.024 |
|  | Higher education (college or above degree) | 0.61 | 0.42, 0.90 | 0.013 | 0.53 | 0.37, 0.76 | 0.001 |
| 60-min Physical Activity | No | 1 |  |  | 1 |  |  |
|  | 1-2 days/week | 0.80 | 0.64, 1.00 | 0.049 | 0.82 | 0.63, 1.06 | 0.121 |
|  | 3-4 days/week | 0.59 | 0.46, 0.76 | <0.001 | 0.76 | 0.59, 0.99 | 0.039 |
|  | 5-7 days/week | 0.70 | 0.54, 0.91 | 0.008 | 0.87 | 0.68, 1.11 | 0.267 |
| Junk food Consumption | No | 1 |  |  | 1 |  |  |
|  | 1-2 days/week | 1.05 | 0.87, 1.26 | 0.614 | 1.34 | 0.63, 0.97 | 0.024 |
|  | 3-7 days/week | 2.28 | 1.58, 3.30 | <0.001 | 1.69 | 0.37, 0.76 | 0.001 |
| Sleep duration overnight | Hours/day | 0.91 | 0.85, 0.98 | 0.012 | 0.97 | 0.91, 1.03 | 0.283 |
| Survey Year | 2007 | 1 |  |  | 1 |  |  |
|  | 2012 | 1.26 | 0.90, 1.75 | 0.179 | 0.89 | 0.66, 1.21 | 0.456 |
|  | 2017 | 1.17 | 0.85, 1.61 | 0.326 | 1.09 | 0.84, 1.41 | 0.515 |
|  | 2022 | 1.73 | 1.30, 2.31 | <0.001 | 1.01 | 0.80, 1.27 | 0.953 |
| **Note:**  a The Pseudo R^2^ is 0.03, the AIC is 3226.58, and the BIC is 3329.20.  b The Pseudo R^2^ is 0.02, the AIC is 3522.93, and the BIC is 3624.94. | | | | | | | |

**Supplementary Table 6.** Associations between over-estimation of weight and academic performances (three categories) for girls and boys.

| **Outcome Variable:**  **Academic Performance**^a^ | **Description** | Girls n=3092^b^ | | | Boys n=2983^c^ | | |
| --- | --- | --- | --- | --- | --- | --- | --- |
|  |  | OR | 95% CI | P | OR | 95% CI | P |
| Over-estimation of Weight | No | 1 |  |  | 1 |  |  |
|  | Yes | 1.24 | 1.08, 1.43 | 0.002 | 1.39 | 1.16, 1.66 | <0.001 |
| Age | Years | 1.03 | 0.99, 1.08 | 0.129 | 1.02 | 0.98, 1.06 | 0.356 |
| Parental Marriage Status | Separated/ Single-parent family | 1 |  |  | 1 |  |  |
|  | Nuclear family | 0.90 | 0.72, 1.13 | 0.345 | 0.86 | 0.66, 1.12 | 0.267 |
| Paternal Education | Primary education or below | 1 |  |  | 1 |  |  |
|  | Secondary education | 0.90 | 0.73, 1.11 | 0.344 | 0.85 | 0.68, 1.07 | 0.160 |
|  | Higher education (college or above degree) | 0.76 | 0.56, 1.04 | 0.084 | 0.74 | 0.54, 1.00 | 0.053 |
| Maternal Education | Primary education or below | 1 |  |  | 1 |  |  |
|  | Secondary education | 0.96 | 0.79, 1.16 | 0.653 | 0.79 | 0.65, 0.95 | 0.014 |
|  | Higher education (college or above degree) | 0.61 | 0.45, 0.82 | 0.001 | 0.50 | 0.37, 0.67 | <0.001 |
| 60-min Physical Activity | No | 1 |  |  | 1 |  |  |
|  | 1-2 days/week | 0.86 | 0.71, 1.02 | 0.089 | 0.88 | 0.70, 1.10 | 0.252 |
|  | 3-4 days/week | 0.67 | 0.55, 0.81 | <0.001 | 0.84 | 0.67, 1.05 | 0.119 |
|  | 5-7 days/week | 0.70 | 0.56, 0.87 | 0.001 | 0.91 | 0.73, 1.13 | 0.402 |
| Junk food Consumption | No | 1 |  |  | 1 |  |  |
|  | 1-2 days/week | 1.06 | 0.92, 1.23 | 0.397 | 1.31 | 1.13, 1.52 | <0.001 |
|  | 3-7 days/week | 2.60 | 1.91, 3.55 | <0.001 | 1.66 | 1.22, 2.26 | 0.001 |
| Sleep duration overnight | Hours/day | 0.99 | 0.94, 1.04 | 0.639 | 1.00 | 0.95, 1.06 | 0.905 |
| Survey Year | 2007 | 1 |  |  | 1 |  |  |
|  | 2012 | 1.34 | 1.04, 1.73 | 0.021 | 0.82 | 0.63, 1.05 | 0.116 |
|  | 2017 | 1.27 | 101, 1.60 | 0.045 | 1.01 | 0.81, 1.25 | 0.951 |
|  | 2022 | 1.81 | 1.44, 2.26 | <0.001 | 1.03 | 0.85, 1.24 | 0.788 |
| **Note:**  a The academic performance was categorized into three ordered groups (good, normal, and poor). The estimation was conducted via the Ordered Logit Regression.  b The Pseudo R^2^ is 0.02, the AIC is 6089.75, and the BIC is 6198.41.  c The Pseudo R^2^ is 0.01, the AIC is 6106.17, and the BIC is 6214.19. | | | | | | | |

**Supplementary Table 7.** Associations between under-estimation of weight and academic performance (three categories) for girls and boys.

| **Outcome Variable:**  **Academic Performance**^a^ | **Description** | Girls n=3092^b^ | | | Boys n=2983^c^ | | |
| --- | --- | --- | --- | --- | --- | --- | --- |
|  |  | OR | 95% CI | P | OR | 95% CI | P |
| Under-estimation of Weight | No | 1 |  |  | 1 |  |  |
|  | Yes | 0.78 | 0.64, 0.95 | 0.015 | 0.91 | 0.78, 1.05 | 0.187 |
| Age | Years | 1.03 | 0.99, 1.08 | 0.125 | 1.02 | 0.98, 1.06 | 0.330 |
| Parental Marriage Status | Separated/ Single-parent family | 1 |  |  | 1 |  |  |
|  | Nuclear family | 0.90 | 0.72, 1.12 | 0.350 | 0.86 | 0.66, 1.12 | 0.269 |
| Paternal Education | Primary education or below | 1 |  |  | 1 |  |  |
|  | Secondary education | 0.89 | 0.72, 1.01 | 0.288 | 0.85 | 0.68, 1.06 | 0.146 |
|  | Higher education (college or above degree) | 0.76 | 0.56, 1.03 | 0.076 | 0.73 | 0.54, 1.00 | 0.050 |
| Maternal Education | Primary education or below | 1 |  |  | 1 |  |  |
|  | Secondary education | 0.96 | 0.80, 1.17 | 0.716 | 0.79 | 0.65, 0.95 | 0.013 |
|  | Higher education (college or above degree) | 0.61 | 0.45, 0.83 | 0.001 | 0.50 | 0.37, 0.68 | <0.001 |
| 60-min Physical Activity | No | 1 |  |  | 1 |  |  |
|  | 1-2 days/week | 0.85 | 0.70, 1.02 | 0.076 | 0.87 | 0.69, 1.09 | 0.213 |
|  | 3-4 days/week | 0.67 | 0.55, 0.81 | <0.001 | 0.83 | 0.67, 1.04 | 0.104 |
|  | 5-7 days/week | 0.69 | 0.56, 0.86 | 0.001 | 0.89 | 0.72, 1.11 | 0.300 |
| Junk food Consumption | No | 1 |  |  | 1 |  |  |
|  | 1-2 days/week | 1.05 | 0.91, 1.21 | 0.488 | 1.31 | 1.13, 1.52 | <0.001 |
|  | 3-7 days/week | 2.61 | 1.92, 3.55 | <0.001 | 1.65 | 1.21, 2.25 | 0.001 |
| Sleep duration overnight | Hours/day | 0.99 | 0.93, 1.04 | 0.598 | 1.00 | 0.95, 1.06 | 0.925 |
| Survey Year | 2007 | 1 |  |  | 1 |  |  |
|  | 2012 | 1.36 | 1.05, 1.74 | 0.018 | 0.82 | 0.64, 1.06 | 0.131 |
|  | 2017 | 1.28 | 1.02, 1.62 | 0.036 | 1.00 | 0.80, 1.25 | 0.982 |
|  | 2022 | 1.82 | 1.46, 2.27 | <0.001 | 1.03 | 0.85, 1.25 | 0.782 |
| **Note:**  a The academic performance was categorized into three ordered groups (good, normal, and poor). The estimation was conducted via the Ordered Logit Regression.  b The Pseudo R^2^ is 0.02, the AIC is 6092.84, and the BIC is 6201.50.  c The Pseudo R^2^ is 0.01, the AIC is 6117.57, and the BIC is 6225.58. | | | | | | | |
